# Understanding the Food Insecurity and Coping Strategies of Indigenous Households during COVID-19 Crisis in Chittagong Hill Tracts, Bangladesh: A Qualitative Study

**DOI:** 10.3390/foods11193103

**Published:** 2022-10-05

**Authors:** Md. Salman Sohel, Guoqing Shi, Noshin Tasnim Zaman, Babul Hossain, Md. Halimuzzaman, Tosin Yinka Akintunde, Huicong Liu

**Affiliations:** 1 School of Public Administration, Hohai University, Nanjing 210000, China; 2 Asian Research Center, Hohai University, Nanjing 210000, China; 3 School of Humanities and Social Science, BRAC University, Dhaka 1212, Bangladesh; 4 Management Science and Engineering, Hohai University, Nanjing 210000, China; 5 Department of Business Administration, Royal University of Dhaka, Dhaka 1213, Bangladesh; 6 Department of Sociology, Hohai University, Nanjing 210000, China

**Keywords:** COVID-19, pandemic, food, food insecurity, food consumption behavior, indigenous community, health, adaptation strategies, CHT region, Bangladesh

## Abstract

This study examined the food insecurity and coping mechanisms among the indigenous Bangladeshi population of the Chittagong Hill Tracts (CHT) region to extract empirical evidence on the ongoing discussion on the COVID-19 pandemic-exacerbated food-insecurity situation. The study adopted a qualitative approach by interviewing 60 indigenous households. Data were collected in two phases between 15 June 2020, and 30 July 2021 in Bangladesh’s Chittagong Hill Tracts (CHT) region. Thematic data analyses were performed using the Granheim approach and NVivo-12 software. The authors used Huston’s social–ecological theory to explain the indigenous coping mechanisms. The research evidence revealed that most households experienced challenges over daily foods, manifesting in the decreasing consumption of them, the increased price of food items, a food crisis due to an income shock, malnutrition, the shifting to unhealthy food consumption, starvation and hunger, and food insufficiency, thereby leading to mental stress. This study further revealed that the indigenous population took crucial coping strategies to survive the pandemic. In response to COVID-19, they took loans and borrowed foods, reduced expenses, changed their food habits, avoided nutritional foods, relied on vegetables, sold domestic animals and properties, collected forest and hill foods, and depended on governmental and societal relief. This study also provides the in-depth policy actions for the urgent intervention of government, stakeholders, policymakers, NGOs, and development practitioners to take necessary initiatives to enhance the quality of life of the people that were affected by the post-pandemic recovery period.

## 1. Introduction

The COVID-19 pandemic caused global disruption and public health challenges, thereby leading to the unprecedented loss of life and negatively impacting education, economies, the workplace, and the food system [1]. The impact on the global agricultural systems disrupted food production, supply networks, trade, markets, people’s livelihoods, and food availability [2]. The World Food Program (WFP) reported that around 272 million people worldwide were food insecure in 2020, and that 957 million people in 93 countries lack sufficient food. Recently, pandemic data from WFP revealed that 97 million suffered from extreme food insecurity due to the COVID-19 pandemic [3,4]. According to a new Oxfam research study, 11 people die from hunger and malnutrition every minute, which is relatively higher than the global COVID-19 death rate which was estimated at being seven people per minute [5]. Therefore, food insecurity has become a global concern in developing countries. According to recent estimates, almost 690 million people, or 8.9 percent of the world’s population, are food insecure [6]. A report shows that 381 million people in Asia and 20 percent of African people are malnourished [7].

In retrospect, the COVID-19 pandemic exacerbated global food instability, reduced incomes, and disrupted food supply systems, thereby causing nagging problems in developing regions. Food insecurity exposure, if it is left unimpeded, and its associated elements, hunger and poverty, will continue to drive negative experiences among vulnerable people [8,9]. Evidence from African countries, such as Ghana, Kenya, and Uganda, has highlighted how the COVID-19 pandemic threatened their populations with food shortages [10,11]. A study showed that there was a rise in panic buying and food hoarding due to food availability concerns in Morocco during the pandemic [12]. Another study highlighted how some women consumed unhealthy food and experienced food-related fear and anxiety due to the COVID-19 pandemic in Egypt, Morocco, and Tunisia. Other documentation shows that food shortages were experienced even in resource-rich countries like the United Kingdom [13], Germany [14], the USA [15], Italy [16], and Russia [17]. Moreover, food consumption and food-related behavior were changed due to the pandemic [18].

The magnitude of global food insecurity cannot be downplayed based on pre-COVID-19 outbreak experiences in some countries. According to the national research center in Canada, the indigenous people that are there had a high level of food insecurity before the COVID-19 pandemic [19]. The COVID-19 outbreak exacerbated the food vulnerabilities among the indigenous population, thereby threatening livelihoods and health [20]. The pandemic affected many indigenous people who engaged in traditional vocations, subsistence economies, or worked in the informal sector. Indigenous women faced more dire situations, as they were often the primary providers of food and sustenance for their families [21]. As the COVID-19 crisis and its associated consequences unfolded globally, roughly 476 million indigenous and tribal people required urgent intervention [22].

Bangladesh is one of the countries that experienced food insecurity during the COVID-19 pandemic [2]. Bangladesh has only 16,50,159 indigenous people who make up 1% of the country’s total population, living in both the plains and the hills [23]. In particular, the Chittagong Hill Tracts (CHT) area makes up about 10% of the total land area of Bangladesh and it has 13 different indigenous groups that reside within it. Most of these people live in Chakma and Marma, the two largest groups in the CHT, and each village has a unique set of traditions, including languages, clothes, and occupations [24,25]. CHT is Bangladesh’s most disadvantaged and vulnerable region in terms of its geographical location, income, employment level, conflict level, land-grabbing practices, poverty level, health, water and sanitation, education, and infrastructure facilities [26]. The indigenous people have a higher poverty percentage than the country’s other regions. Food insecurity is high in the CHT [27], with 62% of indigenous households consuming fewer than 2122 calories per day and 36% consuming fewer than 1805 calories [28]. These food insecurities among the CHT and the COVID-19 food crisis made the UN Food and Agriculture Organization (FAO) refer to the situation as a “crisis within a crisis” [29].

In scope, this research extends the evidence on COVID-19-induced food insecurity among indigenous people in Bangladesh as no study has been conducted among this indigenous population. The relevance of this study is multifaceted, as it presents evidence of food insecurity among indigenous groups during the COVID-19 emergencies.

In particular, this study conceptualized two research questions:

RQ_1_: How did COVID-19-induced food insecurity impact indigenous households in the CHT region, in Bangladesh?

RQ_2_: What coping strategies did indigenous households adopt to survive food insecurity during the COVID-19 pandemic?

After reviewing many pieces of literature, we noticed that some studies had documented COVID-19-induced food insecurity among non-indigenous people in Bangladesh. Until now, no research has been conducted on examining the food insecurity circumstance of indigenous people living in isolated hilly locations in the CHT region, Bangladesh. In this situation, this study intends to address this gap by investigating the COVID-19-induced food insecurity statuses of indigenous households and the coping mechanisms that they used to cope with this pandemic.

Thus, this article can serve as the foundation for further research and broaden the ongoing discussion of the COVD-19-impact on the indigenous people worldwide because there is so little attention and academic scholarship in this field. Overall, the study mapped out potential interventions through policies to alleviate the food crisis among the vulnerable population. Moreover, the findings will be an essential guiding principle for the academicians, policymakers, aid organizations, and development practitioners to prepare sustainable and emergency development policies for the vulnerable indigenous population in Bangladesh and beyond. 

## 2. Literature Review and Theoretical Framework

The marginalized community of Bangladesh experienced food insecurity and associated complications due to the COVID-19 pandemic [30]. Subsequently, there was increased research on food insecurity in Bangladesh. Table 1 provides a tabular literature review of COVID-19 research on food security in Bangladesh. 

Table 2 shows a tabular literature review of COVID-19-induced impact research on indigenous communities worldwide. We found a study that investigated COVID-19 awareness, social responses, and the pandemic’s consequences, such as the prevalence of depression, anxiety, stress, and poor quality of life among indigenous people in Bangladesh [31].

Some theorists have given socio–ecological models for the different contexts. For example, the ecological systems theory that was developed by Bronfenbrenner is one of the most widely recognized theories that is used to explain how social settings affect human development. Similarly, the CDC has provided “The Social-Ecological Model” for stopping violence from occurring. On the other hand, Huston offered social–ecological model to understand marriages and intimate unions. The evidence suggests that this social–ecological theory is widespread, particularly in the social science discipline. For instance, Salin explored Finland’s family coping mechanisms during the pandemic-induced lockdown using this social–ecology theory [48]. Helms used this theory in the Mexican context [49]. Indeed, this theory has already proven useful in the field of social science in the description of several phenomena. We found that this theory is most suitable for our study. Therefore, we used the social–ecological theory to some extent in this work as a theoretical basis for better understanding our respondents’ household adaptation strategies during the pandemic.

**Figure 1:**
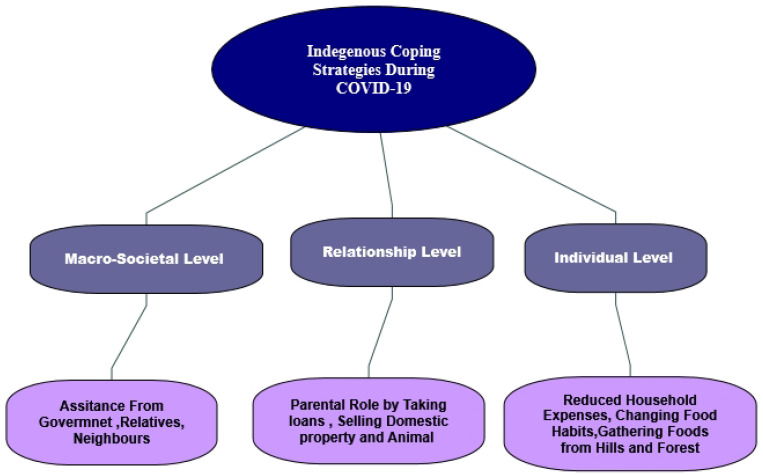
Indigenous household coping strategies through Huston’s social–ecological theory.

In adopting the social–ecological theory, three levels of analysis were conceived and characterized to appraise the coping mechanisms from a broad perspective. These three indicators were identified as group, interpersonal relationships, and individual coping approaches. The group indicators were considered as macro-societal factors through the lens of official and unofficial support for vulnerable communities. We projected that vulnerable indigenous households received societal support from official and unofficial sources through humanitarian aid. The second level of this theory explored interpersonal relationships, such as that which is obtainable from families, neighbors, and friends. This family level supports the efforts of the parents or the heads of households in providing food for their children. Sometimes, parents sourced loans from friends or sold properties to cater to the household’s needs. Individual-level coping techniques supported the understanding of individual coping strategies. It is projected that the household members individually consumed less food, reduced their expenses, changed their food habits, and gathered foods from forests/hills to survive and reduce the household burden. Figure 1 indicates the indigenous household coping strategies through Huston’s social–ecological theory.

## 3. Materials and Methods

### 3.1. Ethical Approval

The study was approved by Ethical Review Committee, School of Public Administration, Hohai University, Nanjing-21000, China, (Protocol No. HHUSPA2021009). The study was also supported by the “Research Evaluation Board of Centre for Advanced Social Research,” in Bangladesh (Protocol number- CRAS20210802). The identity of the respondent was treated as anonymous. Prior approval was taken from the participants before each interview was conducted, and they had the right to withdraw their participation at any time.

### 3.2. Study Area and Location

**Figure 2:**
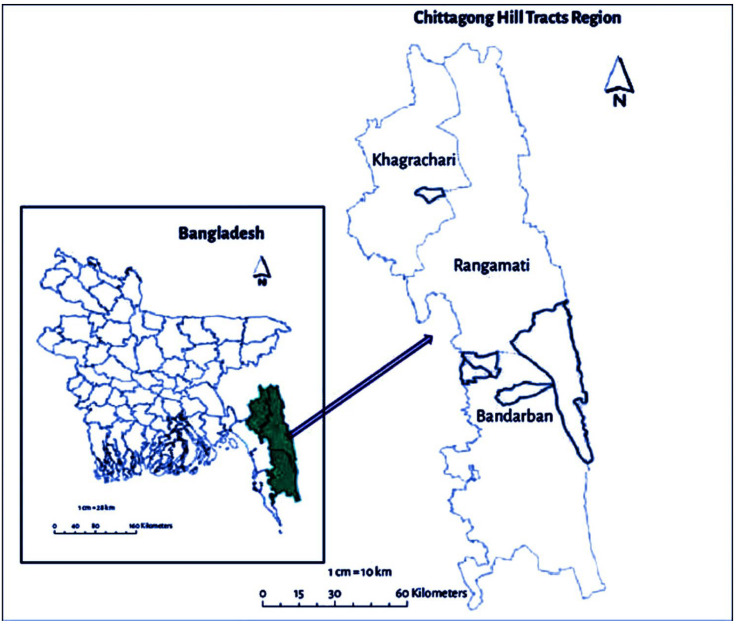
Location of the study area.

The CHT comprises three hill districts: Rangamati, Khagrachari, and Bandarban, which cover 10% of Bangladesh’s total land area. It is bordered on the north by the Indian state of Tripura, on the south by the district of Bandarban, on the east by the state of Mizoram and the Myanmar state of Chin, and on the west by the districts of Khagrachari and Chittagong. It has a subtropical climate, with an annual temperature range of 10 °C to 35 °C and an average rainfall of 2500 mm [50]. We purposively collected data from these three hill districts Rangamati, Khagrachari, and Bandarban. The study area is shown in Figure 2.

### 3.3. Sample Size, Data Collection, and Instruments

A sample size of 10 is suitable for qualitative studies of homogeneous subjects [51]. Twenty respondents could help the researchers to conduct a qualitative study [52]. However, we chose a non-probability purposive sampling technique for selecting the participants. Purposive sampling is a non-probability approach that is often called judgmental sampling, selective or subjective sampling, in which the sample’s chosen components are picked according to the researcher’s sound judgement [53]. Using this approach, the researcher selects the participants who provide the best information for particular issues [54]. To better grasp the challenges of the research subjects, we used a semi-structured questionnaire to collect in-depth information that was based on participants’ experiences [55]. Furthermore, the semi-structured interview technique investigates the more specific details on the research question [56]. Purposively, we selected the household head to be the respondent for this study because the household head has the best knowledge of their family’s affairs.

The CHT consists of three hill districts. We collected data from these three districts. Twenty in-depth interviews were collected from Rangamati, 20 were collected from Khagrachari, and 20 were collected from the Bandarban district. Initially, we conducted eight in-depth interviews for retrospection and further questionnaire development. Afterward, the interview questions were reconstructed to reflect the context of the indigenous community in CHT Bangladesh. We conducted total of 60 face-to-face in-depth interviews with the household heads. Data were collected in two phases: 15 June 2020 and 30 July 2021.

The 1st phase of the data collection was carried out after the first nationwide lockdown (GoB declared the first nationwide lockdown: this lasted from 26 March 2020 to 30 May). We collected 30 in-depth interviews in this 1st phase. Following the first interviews, the interview guideline was, again, refined. The 2nd phase of the data collection phase was carried out after the 2nd nationwide lockdown (GoB declared the second nationwide lockdown: this lasted from 4 April 2021 to 26 July 2021). We completed a second interview round after nine months. In this 2nd phase, we recruited 30 participants for the interviews. The interviewers used facemasks during the interviews and maintained a 3m distance from the interviewees due to the risk of COVID-19 infection. The in-depth interviews were recorded on a mobile device and they lasted between 32 and 55 min. The interviewers took note of the participants’ attitudes, expressions, and voice tones to carefully reflect them in the transcription.

### 3.4. Data Analysis Procedures, Approaches of Taking Measurements, and Coding

The interview transcripts were coded thematically, classified, and organized using the Nvivo-12 software. Data were collected and processed with code numbers in a separate file which were classified by occupation, compiled, and subsequently triangulated according to their nature, type, and characteristics. We used a multiple triangulation approach to ensure that the data’s quality, validity, and reliability was high [57].

The authors followed Granheim and Lundman’s approach [58] to conduct the data analysis, processing, and coding. This data analysis approach is widely used in social science and public health research. Fundamentally, the data analysis was primarily guided by the content analysis methodology that was suggested by Graneheim and Lundman. According to Graneheim and Landman’s explanation, qualitative content analytical approaches concentrate on analyzing a text’s explicit or manifest content and interpretations of its ‘latent content’. Table 3 indicates the data analysis procedures that were used.

**Figure 3:**
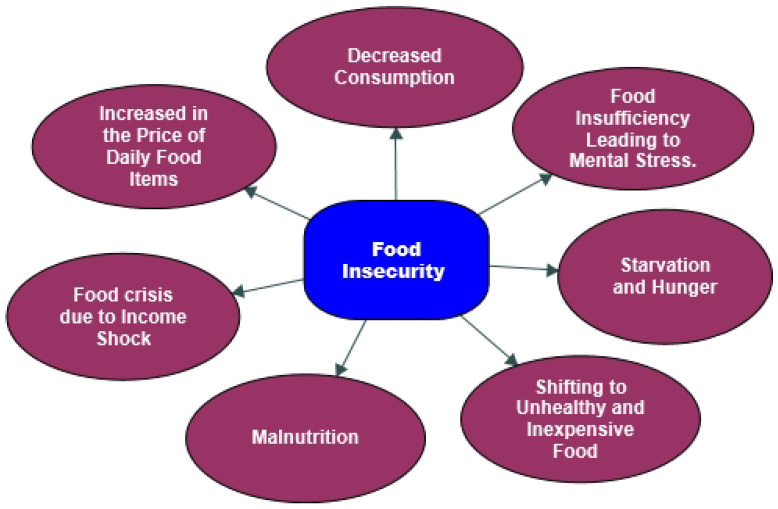
Thematic issues of food insecurity during COVID-19.

According to Robert K. Yin, any chosen quotations or extracts must support the interpretations and justifications that are made [59]. As there are no rules dictating how many there should be or how lengthy the quotations or extracts should be, rather, the researcher must decide how to use them, when they are suitable, and how to ensure that the quotations match the context [60]. It depends on the interpretation and context of the theme or sub-theme. The illustrative quotations or those that have been written verbatim provide strong evidence of the researcher’s understanding of the population’s feelings. Thus, the authors chose robust quotations that fundamentally represent and fit our narrated context. These quotations justify our explanations in the result section—the following figure, Figure 3, was developed by the authors using the field data.

Subsequently, two central themes, food insecurity and coping strategies, were derived from the qualitative data analysis using NVivo-12 software (See Table 4). The food insecurity theme produced the highest reference code value from the NVivo-12 data with there being seven sub-themes in this, while the coping strategy theme had the second-highest reference code value with there being six sub-themes in this.

## 4. Results

Table 5 summarizes the demographic profile of the research participants. The age range of the participants was between 20 and 70, with there being 39 male and 21 female participants. Additionally, most of the participants were uneducated, with the highest level of education being high school. All of the respondents were married, while 14 of the participants were widows. One-third of the indigenous population earns between 7000 BDT to 10,000 BDT ($81 to $116) monthly. Meanwhile, 13 indigenous households earn below 6000 BDT ($69) monthly. Collectively, nine households can earn between 16,000 BDT-20,000 BDT ($186 to $232) monthly. However, most of the respondents who were recruited for this study are from the Marma and Tripura ethnic community

### 4.1. Food Insecurity

The COVID-19 pandemic created significant challenges globally. In Bangladesh, particularly in the Hill Tracts region, indigenous people lost their jobs and experienced severe starvation which was caused by the COVID-19 outbreak. Additionally, official and non-governmental support programs were hampered due to these people being in a remote and hilly area, thereby causing the poor coverage of relief intervention support in the CHT region.

The exposition from the thematic analysis revealed seven sub-themes: the decreased consumption of food, an increase in the price of daily food items, a food crisis due to income shock, malnutrition, the shifting to unhealthy and inexpensive food consumption, starvation and hunger, and food insufficiency, thereby leading to mental stress.

#### 4.1.1. Decreased Food Consumption

The indigenous community suffered from having limited resources to support their livelihood. Before the COVID-19 crisis, the residents of the Chittagong Hill Tracts region were deprived of basic facilities in comparison to other regions in Bangladesh due to its geographical positioning. The COVID-19 lockdown caused industries, markets, and offices to shut down, thereby causing hindrances in purchasing daily necessities. A reduction in the number of household income-generating opportunities further negated these identified social problems [61]. The indigenous people experienced decreasing levels of food consumption.

*“We had to reduce our consumption due to the pandemic. We had no income and sat idly at home during the lockdowns. We ate what we had at home” *

*(Interviewee #10)*

Before the pandemic, indigenous people had income-generating opportunities to be able to afford three meals daily. However, the COVID-19 outbreak and the strict lockdown measures complicated this experience, and thus, they limited their ability to eat more than two meals daily. In addition, farmers’ local agricultural activities could not continue due to isolation and travel restrictions [62].

*“Before the lockdown, I had access to three meals daily. During the 2nd lockdown, food availability was limited. I eat twice a day, one kg of pulses. Markets were inaccessible for buying or selling goods. When there are opportunities to sell, it is impossible to get a fair price. Goods are sold at low prices” *

*(Interviewee #6)*

#### 4.1.2. The Increased Price of Food Items

The study found that participants had difficulty purchasing essential goods due to there being increased prices, high demands, and low supplies of them [63]. The COVID-19 measures of the lockdown and the stoppage of inter-city movement further complicated the situation, but government interventions brought some relief during the second lockdown.

*“When the government imposed the 1st nationwide lockdown, there was a massive food crisis in the market with a limited number of goods. This imposition caused an increase in foods price relative to food supplies” *

*(Interviewee #16)*

Another factor which was attributed to the high food commodity price was the transportation cost of it [64]. Some business owners hired high-cost vehicles for transferring goods to continue their daily transactions. To break even, they had to increase the price of these goods.

*“I have a grocery shop. All types of transport services were shut down due to the lockdown. I have to contact a local delivery agency to hire a vehicle to transport goods. They overcharged us for their services” *

*(Interviewee #5)*

The laborers had difficulties accessing the market at certain times as they could not abandon their work. Additionally, there was visible marginalization which occurred as some stores secretly opened to their customers in order to sell their goods to them. So, they pay a high price for the goods that they purchased.

*“We had no oil in the house; cooking was impossible. Grocery stores open at certain times of the day. I couldn’t go to the store due to my busy schedule. Also, the shops were closed, so we had to buy inflated goods at a high price secretly. Isn’t that a problem?” *

*(Interviewee 1)*

#### 4.1.3. Food Crisis due to Income Shock

The income shock that many people experienced ultimately led to the food crisis, which caused unsavory experiences among indigenous groups. A study articulated the effects of food prices and income shocks on rural communities’ food security and economic well-being. Thus, households that experienced income shocks were more likely to be food insecure [65]. Due to the inaccessibility of food supplies, people reduced their budget and consumption of food. The situation worsened during the first phase of the COVID-19 lockdown.

*“My son worked as a store manager in Dhaka. He lost his job due to the lockdown. We worried about this. We can hardly afford food due to our poor economic condition”*

*(Interviewee #23)*

Small businesses and enterprises experienced a significant hit. Most of the small businesses were forcefully closed due to the lockdown, while one-third functioned at a sub-optimal capacity during the early stages of the lockdown [66]. Therefore, business owners and traders could not buy or sell goods.

*“My trade business has been closed since the first lockdown limiting income-earning capacity. I have been doing this job since childhood. Now, I cannot continue the business. The job market is failing, and I have been living on my savings. Before the lockdown, I ate fish and meat almost every day. Now I cannot buy fish anymore. I have a meager budget for food” *

*(Interviewee #8)*

#### 4.1.4. Malnutrition

Malnutrition has always been a challenge in Bangladesh due to the population explosion that occurred and the multidimensionality of poverty. Three CHT districts (Rangamati, Khagrachari, and Bandarban) have a malnutrition rate of 48 percent with the prevalence of this being among mothers and children [67]. The COVID-19 pandemic created more complications and pushed them towards the extremities of malnutrition. The high price of goods, the restrictions on trade and transportation, and the closure of markets, groceries, and industries negatively affected consumption rates. The indigenous people had to rely on cheap foods with low nutritional values rather than consuming vegetables or protein-rich food.

*“Police did not allow us to go outside nor go to work. I ate pulses for many days and could not afford nutritious food because I had no money” *

*(Interviewee #24)*

Recently, the World Bank published that a reduced calorie intake and poor nutrition among people can jeopardize the progress in reducing the rate of poverty and may have long-term consequences for children’s growth and development [68]. From our findings, the indigenous women in CHT had difficulty accessing nutritious food to feed their children. This problem also affected breastfeeding among mothers. Starvation led to malnutrition for both the mother and child. One of the respondents said, with tears in their eyes:

*“My child often cried continuously due to inconsistent breastfeeding. No milk didn’t come in my breast because I was starved very often” *

*(Interviewee #19)*

#### 4.1.5. Shifting to Cheap and Unhealthy Food

According to the World Bank, there is a shortage in the food supply in 89 countries, thereby impacting individual consumption levels [68]. Populations that are dealing with food shortages often resolve to food substitutes for their survival. The indigenous people had to consume spoiled food or waste. This situation represents the magnitude of food insecurity among the indigenous people during the COVID-19 lockdown.

*“This is so embarrassing, but the truth is that I had no money. So, I bought rotten vegetables and rice. During the whole lockdown, I preferred cheap food.” *

*(Interviewee #7)*

The lockdowns that were instated worsened indigenous life [69]. The food prices became high during the lockdown. So, people purchased and consumed rotten food because it was affordable and accessible.

*“Cheap and semi-rotten foods are less expensive than fresh foods. It is the perfect option for me in this crisis. I do not have enough money or savings. Hence, the only option was to survive by eating semi-rotten food. It does not matter whether it is wrong or good for health” *

*(Interviewee #9)*

The study results also revealed that food such as potatoes, snails, beans, etc., were accessible to poor, indigenous people.

*“Mashed potatoes, snails, and mussels were in my food chart during the lockdown because it was cheap“ *

*(Interviewee #33)*

#### 4.1.6. Starvation and Hunger

During the COVID-19 outbreak, the women who were the heads of the indigenous households experienced severe starvation. The travel restrictions in many districts and territories distorted the economic activities of the indigenous people.

*“My husband died two years ago. Now, I have to work for survival. But I can only afford to eat a simple morning breakfast during the COVID-19 quarantine. I cannot afford to have proper three times meals daily. I am worried about starvation” *

*(Interviewee #50)*

Additionally, more evidence emerged that some indigenous people resisted the sensation of hunger and preferred to drink water. This action is primarily due to the availability of water in the region. One of the participants who drank water to survive had a low financial capacity to afford food.

*“I can’t afford to eat properly. Sometimes, particularly in the first lockdown, I just drink water to fill my stomach “ *

*(Interviewee #48)*

Around 10% of the world’s population, or up to 811 million people, usually go to sleep hungry. More than 48 million people are facing alarming levels of hunger, with the threat of experiencing acute malnutrition, starvation, and death, according to the World Food Programme (WFP) [70]. For the most of the vulnerable people, the COVID-19 pandemic, armed conflicts, and climate change have caused unprecedented predicaments for them [71]. Similarly, the CHT region has been impacted by massive armed conflict and climate-induced disasters. Moreover, the acute food crises, such as hunger and starvation, are still prevalent in hilly areas [72]. The COVID-19 crisis distorted the indigenous livelihood system, forcing them to experience severe food shortages. We obtained the reports of such scenarios occurring from the participants during the interview sessions.

*“Most tribal families in the hill areas are poor and have more or fewer food problems. But COVID-19 exacerbated the food crisis. In my case, some days, I remained starved and hungry during the lockdown. I cannot request food from anyone. I feel shy. That’s why I suffered more” *

*(Interviewee # 29)*

#### 4.1.7. Food Insufficiency Leads to Mental Stress

Food insecurity is linked to elevated levels of anxiety and stress [73]. The psychological vulnerabilities that were experienced by the research participants raised tensions that were associated with food insufficiency during the pandemic. Several interviewees discussed worrying about food, while also being concerned about covering other living expenses and repaying loans.

*“Lockdowns have destroyed our normal life. I have no income, employment, and no food in my house. I don’t bother about myself but worry about my kids and wife. Day by day, the mental stress is increasing” *

*(Interviewee# 50)*

The pandemic increased both the rates of food insecurity and poor mental health. According to the data, indigenous people have an undue risk of experiencing mental health issues, worldwide [25]. Many of the interviewees expressed dissatisfaction with their household food situation.

*“Sometimes I feel like committing suicide. The lockdown sent me into depression, especially when I had to cater to a family of five with no husband to provide support. Before the lockdown, I used to sell street foods. But now I can’t do it because of the government’s strict restrictions. My child cries for food. I don’t know when the lockdown will be lifted. We live in a remote hilly, and hard-to-reach area. It is challenging to receive food assistance here” *

*(Interviewee #23)*

### 4.2. Indigenous Coping Strategies during COVID-19

Bangladesh is one of the world’s most vulnerable countries, with millions of people being impacted by natural disasters [74,75]. Thousands of people experience storms, cyclones, drought, erosion, heavy rain, tornadoes, landslides, and flooding, yearly. Therefore, coping strategies and resilience play vital roles in dealing with any disaster, especially with the recurring disasters in Bangladesh [76,77]. Socio-cultural, economic, and structural elements shape the CHT region’s indigenous coping techniques. Based on the interviews and field observations, the indigenous people have particular aptitudes to deal with the devastating COVID-19 situation. The study found that the indigenous people in the Hill Tracts region employed primitive coping mechanisms to survive the COVID-19-induced food crisis. Some of their coping mechanisms follow the premises of Huston’s social–ecological theory [78]. According to the study’s findings, the indigenous people took loans, sold domestic animals and properties, reduced their budgets, decreased their consumption of food, changed their food habits, collected foodstuffs from the forest, and received support from relatives, neighbors, and the union council.

**Figure 4:**
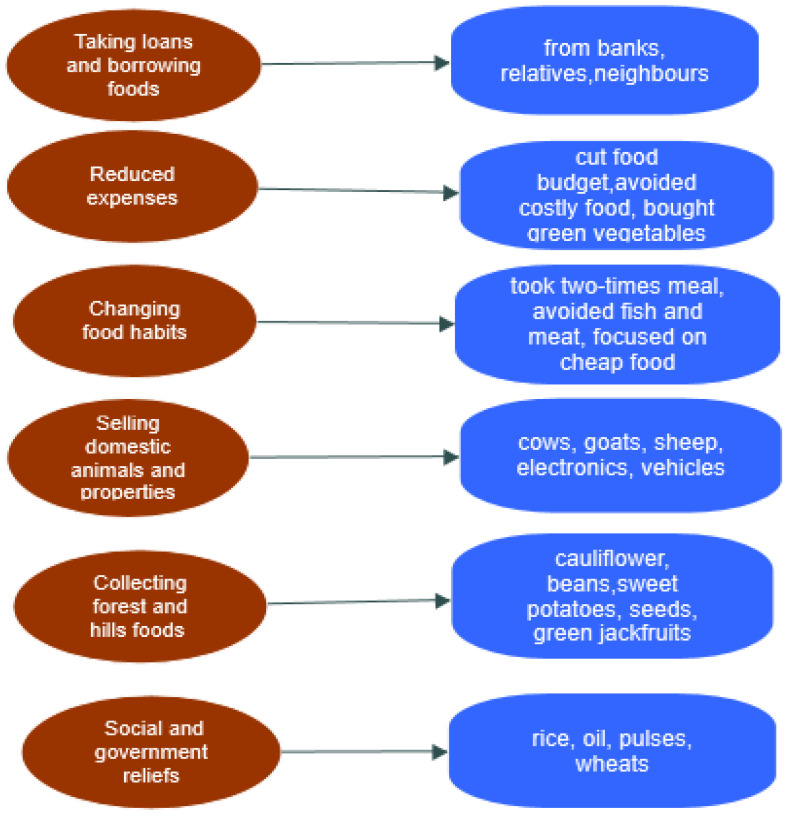
Indigenous coping strategies.

Based on the theoretical premises of the study, it is confirmed that the coping mechanisms among the indigenous groups follow the trajectory of individual, interpersonal, community, and external (organizational) support at various stages of the COVID-19 public health crisis. This evidence highlights how they survived, as individuals, and when they were in need, they sought help through interactions with neighbors or relatives (interpersonal relationships). Similarly, external agencies and institutional interventions provided the indigenous population with relief at different pandemic stages. Figure 4 shows the coping strategies of the indigenous households during the COVID-19-induced lockdown based on the social–ecological theory.

#### 4.2.1. Taking Loans and Borrowing Foods

The individuals borrowed money to stabilize their ability to cope with income shocks during the pandemic [11]. Similarly, we found that the indigenous households took loans from banks, organizations, friends, and relatives to cope with the loss of jobs and insufficient relief which was provided during the COVID-19 lockdown. Huston categorized this approach in the relationship level, describing the parents’ role in providing support [78].

*“I had to borrow one lakhs money from Janata bank and relatives. Having no income for such a long-time (6/7 months), I had to take loans to maintain the family. But I have not been able to repay the loan with reduced income. I spend a large amount of money monthly. It is challenging to repay loans because of the daily expenses” *

*(Interviewee #36)*

The condition of borrowing money by the indigenous people is attributed to their resilience to survive in the face of having a low income and strained livelihoods. An interviewee showed extreme emotion while recounting his income and food situation, and he mentioned how he had to source food from neighbors.

*“I had no income, no money, and food at home. I stayed for a few days without food, and didn’t want to die because my wife and three children rely on me. So, I had to borrow food from my relatives” *

*(Interviewee #39)*

Meanwhile, some indigenous people had no opportunity to borrow foodstuffs from neighbors since everyone that they knew faced similar financial and food crises. They resolved to borrow food from others without the assurance of repayments.

*“I approached some people to borrow foodstuff, but none of them were able to give me. I hardly borrow paddy from a neighbor. But now, I am tense about paying them back. I am not certain my situation can improve in the future” *

*(Interviewee #21)*

#### 4.2.2. Reducing Expenses

In his theory, Huston explained that individual-level coping strategies are an individualistic attempt to survive difficult situations [78]. COVID-19 adversely impacted people’s consumption of food and their daily expenses. Before the pandemic, indigenous people of moderate income could afford to live an average lifestyle. However, many indigenous households struggled to afford one meal daily during the lockdown [79]. As a result, they had to take the initiative to cut down on their budgets. The evidence in our research shows that most of the indigenous people with no job or no savings had to cut down on their food expenditure.

*“I had to reduce my budget for food because I could not afford three meals daily” *

*(Interviewee #23)*

The study’s results also show that the indigenous people had to change their food habits to resonate with their low income.

*“Usually, we eat fish and meat at least two days a week. Now, to cope with the food situation, I do not buy fish anymore” *

*(Interviewee #8)*

One of the participants recounted the situation surrounding access to green vegetables. In the Hill Tracts areas, green vegetables are usually available everywhere. Many indigenous people engaged in rubber planting, vegetable gardening, and Jhum cultivation. Most of the families that live there own small to medium gardens in their homes and sell the produce from these at lower prices in the market. Green vegetables were quite accessible for many of them.

*“My income is quite low. I could only afford green vegetables and no other food items” *

*(Interviewee #60)*

#### 4.2.3. Changing Food Habits

Changing their food habits was one of the indigenous household coping strategies, which is an individualist approach which resonates with Huston’s individual level premise [78]. The eating behaviors of these households changed massively during the lockdown. For example, those who ate three times daily had to cut this down to two meals a day or less.

*“Before the outbreak, I used to eat three times, and now I have to eat twice a day one kg of pulses” *

*(Interviewee #57)*

Most of the indigenous people in CHT engaged in agricultural activities, small businesses, homestead gardening, or practiced street fruit selling as a livelihood. Consequently, low-paid jobs were their only means to make ends meet. With the massive collapse in employment opportunities, many of them had no choice but to engage in low-income jobs for their survival.

*“Before lockdown, I could purchase meat. Now, meat and Fish are unaffordable with my current income. I mostly now eat hill potatoes or pulses with rice twice daily during the lockdown“ *

*(Interviewee #2)*

#### 4.2.4. Selling Domestic Animals and Property

Selling domesticated animals to survive starvation was a significant food-seeking behavior [80]. The indigenous people also sold cows, chickens, goats, and other livestock for quick access to cash. This coping strategy is similar to Huston’s relationship level [78]. However, one of the research participants had to sell her domestic animals because she had to repay loans.

*“Yes, I had to sell chickens and goats since I was unable to repay all the loans” *

*(Interviewee #17)*

The enormity of the food insecurity that was experienced caused the indigenous people to sell off significant properties to ensure that they had food stability.

*“The only way to earn money is to drive a tomtom (autorickshaw). I had to sell my tomtom. Before the pandemic, I had three tomtoms; now, there is only one. I had to sell the other two because of the food crisis” *

*(Interviewee #56)*

The prices of the food items caused significant setbacks in alleviating the burden of food crises among the population. A participant mentioned that he sold his cows and goats at reduced prices.

*“We had to sell our cows and goat. Due to the food shortage, we had to sell them cheaply” *

*(Interviewee #38)*

#### 4.2.5. Collecting Forest and Hills Foods

CHT is regarded as a bio-cultural diversity hotspot due to the abundance of natural resources and cultural diversity within it. The indigenous people suffered a lot when the government declared strict, nationwide lockdowns. In this situation, they used to collect food from the hills and forests through the Pahari (hill) food network. We found that this Pahari (hill) food network is one of the vital coping strategies for indigenous people who are aiming to reduce their level of food insecurity to some extent [81]. Indeed, historically, the indigenous population in CHT has depended on the forest’s natural resources for their livelihood [82,83].

*“I collected cauliflowers (a popular hill vegetable) from local forests as it was an easy food option” *

*(Interviewee #6)*

We asked the participants about the distance from their homes to the forest as the Chittagong, Rangamati, and Khagrachhori districts contain many hills and mountains. There are significant disadvantages to the transportation of food since there are poor road networks in remote areas.

*“Our homes are located near the hills. Therefore, my brother and I gathered beans and sweet potatoes daily since it was tough for us to go to the market or afford high-priced food” *

*(Interviewee #33)*

Many indigenous people depended on famous heritage sites and temples to sell their products. All of the tourist centers were closed during the lockdown. Collecting food from the forest minimized the effects of the food crisis and this was used as a coping strategy.

*“I used to sell clothes at my shop near Jogonnath Mondir(temple). But, after the lockdown, we had to shut down our shops and could not get out for months. I collected beans, green jack fruits, and seeds from the forest” *

*(Interviewee #22)*

#### 4.2.6. Social and Governmental Reliefs

While facing the COVID-19 health crisis backlash, the government and institutional support opportunities that were offered were crucial to their survival. Thus, the government, organizations, local government, and affluent individuals provided support through aid and relief. This coping method corresponds to Huston’s macro-societal level. One of the interviewees reported a positive review about receiving help from different sources. Additionally, he mentioned that there were wealthy locals who helped him, personally.

*“They couldn’t provide everything we needed, but they tangibly supported us. I am satisfied. I don’t know the names of the organization, but they supported us along with some rich individuals” *

*(Interviewee #60)*

Organizational support was delivered in the form of donations. The study shows that offerings were conducted in an orderly fashion, but they were limited. The indigenous received some relief from both social and governmental agencies.

*“The chairman donated rice, pulses, and oil twice during the lockdowns. He donated in the first lockdown and second lockdown. They engaged the military in arranging the market in the stadium. Army personnel gave me the slip, and I went there to get rice, pulses, arums, and cucumber” *

*(Interviewee #57)*

Besides, there was support from the administrators and influential people. Indigenous people received food from their neighbors, relatives, and volunteers. Many young volunteers took the initiative to help the affected and poor households. They provided food assistance, along with government officials and village police.

*“I was sitting in the yard here. A few Tripura young people came and gave us 5 kg of cooked rice once. Our relatives gave some rice, pulses, and oil. We got those twice during the lockdowns”.*

*(Interviewee #3)*

## 5. Discussion

The social vulnerabilities that were caused by the COVID-19 pandemic caused unemployment and reduced incomes for indigenous people, thereby causing food insecurity situations, in which they had to survive through any means that were possible. The research evidence shows that the indigenous groups of CHT experienced worse situations when these were compared to those of the indigenous mountain communities of Peru, Kenya, Papua New Guinea, Bhutan, and Tajikistan. These indigenous mountain communities utilized biodiverse, regional, and regenerative food systems [84]. Food insecurity is associated with a diminished income and a decreased consumption of food. This study shows that the indigenous people decreased their consumption of food due to them experiencing financial constraints. Leweniqila and Vunibola reported similar findings among the indigenous Fijian communities. The Fijian people are primarily dependent on the tourism sector [85]. However, the COVID-19 pandemic affected their means of sustaining their livelihood. As a result, the indigenous people experienced a significant income shock, thereby resulting in lower-quality food consumption. Another study focused on South Asian rural families. It explained that nearly half of the population lives in extreme poverty due to the impact of job losses, with them experiencing food shortages [84].

Our study found another reason for food insecurity which is the occurrence of price hikes. A decline in the number of commodities increased the demand for goods and their prices. We obtained similarity in the results that were based on a study of Iran [86]. This study indicates that a fall in goods production leads to higher prices for food and commodities. Ultimately, there will be a decrease in the people’s buying capacity, while there is an increase in family spending during the pandemic. Food production also decreased, which disrupted food supplies during the global pandemic. Additionally, the food supply chain interruption occurred due to the closure of international borders. This influenced food availability [87]. Consequently, the issue of food supply has grown more problematic in the market [88], directly affecting household food security. Furthermore, physically and financially, food availability became challenging throughout the pandemic [89]. When the government ordered that the lockdown be thoroughly enforced, thus preventing all modes of transportation from moving food, the unemployment, the high price of goods, and the unavailability of healthy foods that occurred as a result negatively affected the indigenous group’s consumption behaviors. They struggled to buy necessary food items. 

Similarly, the COVID-19 lockdowns caused social isolation that impacted lifestyles, including decreased physical movement and changes in eating habits. The indigenous groups changed their food habits to cope with food insecurity. The governmental imposition of the lockdowns halted all of the economic activities, including transportation and employment, thereby causing an increase in goods prices. Due to the COVID-19 outbreak, the ethnic community of Batwa in Rwanda faced an income shock as their traditional means of sustaining their livelihood could no longer sustain them [90].

Adaptation mechanisms are essential for having resilience to any natural disaster [91]. The data revealed that the indigenous people followed some crucial measures to fight the pandemic. Most of the indigenous people took loans and borrowed food from multiple sources during the crisis. As the COVID-19 response measures caused unemployment and reduced incomes for the indigenous people, many people reduced the number of meal items and the amount of food that they ate or they skipped meals due to them having insufficient finances [92]. The indigenous groups adopted extreme measures by consuming cheap vegetables instead of expensive food items like meat and fish, and compromised by selling off their assets [80]. These extremities were also witnessed among Thunder Bay’s ethnic group, the Wayan community, who live in Brazil [93]. To meet the food challenges that they faced, our respondents grew different vegetables like jackfruits, beans, potatoes, cabbage, etc. Most of the vegetables that they collected were from their garden or local trees [92]. At the same time, the traditional harvesting practices by indigenous people in Australia and Aotearoa were also adversely impacted [93,94]. The CHT indigenous people did not obtain enough food to consume. They had no choice but to eat low-cost items rather than nutritious food like green vegetables or those containing protein. In Columbia, the indigenous children were in a high-risk condition because of the food insecurity that they experienced. They faced shocking malnutrition and death at that time [95].

In Bangladesh, most of the indigenous populations who had similar experiences chose the option of eating rotten or cheap food. Galali also revealed that a Kurdish indigenous community included rotten or cheap food in their food menus [96]. Additionally, a study investigated a shift that occurred in food consumption from more expensive sources of calories to less expensive ones [97]. In such a situation as this, the indigenous Bangladeshi people who live in the Chittagong Hill Tracts area experienced significant food insecurity and used various coping measures to survive the pandemic. These coping mechanisms resonate with Huston’s social–ecological theory. The theory expands on the complex connection between individuals, their relationships, and the community setups. The scope of the current research among the indigenous groups addressed family coping mechanisms during the COVID-19 pandemic, food insecurity at the macro-societal level, associations with others, and individual measures that were taken. Huston’s macro-societal level projected that friends, neighbors, and families facilitate social support and group support [78]. The indigenous population exhibited these vital attributes through sourcing foodstuffs from friends/family, neighbors, non-profit organizations, and the government. The relationship-supported coping strategies that we address here mean those which reflect a parental role in the family during the food crisis. In this study, we found that the indigenous parents played a vital role in the continuation of household affairs during the pandemic. Parents sold domestic animals and properties to purchase food. Huston’s third level indicates the individual effort in coping with the food situation. The participants personally took various initiatives to adapt to the food crisis by reducing their expenses and decreasing their consumption of food.

Moreover, the result showed that the respondents received food relief from the social and government agencies during the lockdown. They received rice, pulses, oil, soap, and other forms of tangible support from non-profit organizations, neighbors, and the local government. According to Kaplan, in some European countries, the government distributed stimulus cheques that had a value which was relatively lower than people’s basic income was [98]. Our data indicate that the support of the government and the NGOs which was offered to the vulnerable groups was minimal. The indigenous group of CHT required more intervention to ease the burden of food insecurity that was due to the pandemic.

### 5.1. Policy Recommendations

The evidence is apparent. In such a situation as this, the government should increase their aid and fiscal spending, thereby targeting these vulnerable populations to move forward from the food insecurity issues of the indigenous groups for a post-COVID-19 pandemic redress. Since the evidence from this study can start as a foundation for the government, the policymakers, aid organizations, stakeholders, and associate agencies should adopt this study to understand the scenario of food insecurity among indigenous households during the COVID-19 pandemic, which will help them to prepare welfare policies and interventions for the indigenous community. The food insecurity issue has short and long-term negative impacts on the livelihood of the indigenous population. Government-sponsored humanitarian aid programs, such as food assistance measures, are insufficient to ensure the population’s survival [99]. A wide range of actions are required to address indigenous food issues. The evidence among the research participants identified the failure and collapse of numerous small and medium-sized informal businesses due to the COVID-19 financial crisis. For the interest and survival of the indigenous groups, the government should provide cash support to those who wish to continue their profession to alleviate the rate of unemployment and to ensure household resilience. The associated income shock that was experienced by the indigenous group from the loss of their small and medium businesses should be alleviated by the government through cash payments for them to manage these small businesses and their household activities. The government should expand the employment opportunities through local government poverty-reduction projects such as “Food in exchange for work (KABIKHA” and “money in exchange for work (KABITA).” These initiatives can reduce rates of malnutrition, poverty, and unemployment and improve household food consumption.

The price hike that occurred for food commodities and services ushered in difficulties in buying and selling with the low-purchasing power of the indigenous group. Governments should manage local indigenous production markets, such as fixing the price of products, purchasing produce from farmers, balancing stocking products, and providing subsidies for fertilizer, seeds, and pesticides. Furthermore, as the livelihood of the CHT regions is primarily dependent on agriculture, the government should compensate the farmers for lost productivity due to the lockdown, particularly for vulnerable indigenous farmers. The supporting of indigenous people who took loans from relatives and banks due to them experiencing massive income shocks and unemployment during the lockdowns is a situation that requires immediate government action. The government should provide loans and credit services to vulnerable people who have businesses and farmers to minimize the burden of preexisting loan repayments. Government should facilitate state finance access by reducing the complexity of the administrative procedures, the requirements, and the length of the processes. Because a few of the research participants received no government support or insufficient support, the government should enlist vulnerable households in social protection and social safety net programs. Lastly, because the research participants revealed that there were transportation constraints during the COVID-19 infection waves, it became imperative for the government to remobilize the transportation system and help the local indigenous people deliver their goods outside of their cities during the COVID-19 crisis. In this case, we suggest that the government utilize the state-owned buses and goods carriers. This might help those who have to travel for work, business, academic, or healthcare purposes to access these essential services and contribute to keeping this country operating during any crisis that is like the COVID-19 pandemic.

### 5.2. Strength and Limitations

The unique, qualitative methodology and study population is the main strength of this paper. This is the first qualitative study in Bangladesh to understand indigenous food insecurity and the coping strategies that were adopted by indigenous households during the COVID-19 lockdown. This study also provides in-depth policy actions for the urgent intervention of government, stakeholders, policymakers, NGOs, and development practitioners to take necessary initiatives to improve the quality of life of the affected population for the COVID-19 pandemic recovery period.

One limitation that is identified in this study is the sample size, which was adopted, which may be subjected to a sampling bias. Similarly, this study did not consider other indigenous groups of Bangladesh’s Mymensingh, Sylhet, and Rajshahi divisions. As a result, the findings of this study may not be generalizable, which allows other researchers to further explore this area by adopting a larger sample size and quantitative approaches.

## 6. Conclusions

The COVID-19-caused pandemic triggered various countries to experience food crises that led to a global imbalance in food accessibility, availability, and in the supply chain. In particular, COVID-19 and its implications have severely affected the poor communities of Bangladesh, including the vulnerable indigenous people in the CHT region. Therefore, this study explores indigenous food insecurity and the coping strategies that were used during the COVID-19 pandemic in the CHT region of Bangladesh. The research evidence in this study consolidated the enormity of the food insecurity that was experienced among the indigenous population of the CHT region in Bangladesh. The general belief among the research participants was that there was a cessation in food production, restricted economic activity, and job losses which were triggered by the COVID-19 emergency, which acted as a barrier to accessing food in sustainable quantities. Consequently, the indigenous population witnessed decreased levels of food consumption, a high cost of foodstuffs, low income and purchasing power, poor nutrition, shifting to unhealthy and low-quality food, starvation and hunger, and mental stress. In response, the indigenous population adopted crucial coping strategies during the COVID-19 lockdowns, such as taking loans, reducing their spending, changing their dietary habits, and selling their personal properties. Some adopted measures like collecting vegetables and foodstuffs from the forest, while some received governmental and societal relief.

The results of this study have significant practical implications for decision-makers to help them to address COVID-19’s impacts on countries’ food insecurity levels. Theoretically, this study contributes to the body of literature in a number of ways, including in the development of critical indicators for assessing food insecurity among indigenous households, the use of qualitative methods to investigate the current COVID-19-related circumstances of indigenous households, and the addition of significant findings for assessing the vulnerability that was brought on by the COVID-19 pandemic.

## Figures and Tables

**Table 1:** Tabular literature review of COVID-19 researches on food security in Bangladesh.

Reference	Methodology and Sample Size	Key Findings	Limitations
[32]	Multivariate Multiple Ordinal Logit Regression, partial least squares path model with n = 540	Higher COVID-19 severity correlating to starvation because of income shock and price hike, and food insecurity affecting purchasing and consuming patterns	Negative weights, multicollinearity, unavailability of global index for model validation, limited use of goodness-of-fit
[33]	OLS approach with n = 50,000	Estimation of the economic damage caused by the COVID-19-induced lockdown and proposed a minimal financial package to maintain food security for Bangladesh’s daily paid workers	Lack of theoretical discussion
[34]	Multi-equation partial equilibrium with rice market data from FY96 to FY20	Decrease in food security, higher import tariff limiting rice supply and stock enhancement strategy mitigating the negative impact	Absence of the influence of economy-wide imbalances on the Bangladeshi rice market, and the dynamics in relation to other agricultural and non-agricultural sectors
[35]	Longitudinal study with 3544 persons Bangladesh, 3685 persons in Kenya, and 3582 persons in Nigeria	Unemployment, shutdown of business, disruption in agricultural activities, price hike, sickness/death, selling assets, extra earning, monetary support, decrease food/non-food intake, savings	No mention about most vulnerable demographic groups, and reasons of inequalities, no discussion whether job loss and business shutdown related to labor demand/supply, no theoretical framework
[36]	Food Insecurity Experience Scale (FIES), linear probability model, 10,000 households	Food insecurity increased dramatically across families and began to impact groups that were in a better situation in the first survey	Phone interview, two study areas, no discussion on the issue of endogeneity under various data and interview constraints, lack of exogenous fluctuations, no theoretical linking
[37]	Descriptive statistics, n = 397	Income shock, unemployment, price hike, reducing grocery shopping, high-cost goods and unhealthy snacks, intaking nutrient food, adopted eggs and dried fish and stored rice, lentils and potatoes	Absence of theoretical framework, missing answers to some questions, the number of participants for different indicators was not the same
[38]	Cross-sectional survey, n = 1876	Decrease in consumption, job lost, or shut down businesses, income shock	Lack of theoretical discussion, short time frame—5 months, online participants, unable to assess any seasonal fluctuation in HFS and HDD, self-reported data by the participants
[2]	Interpretive phenomenological analysis, 21 in-depth interviews and 4 FGDs	Skipping meals, rising prices, and a scarcity of fish, meat, potatoes, and vegetables, chronic nutritional scarcity, hunger, maternal and child malnutrition, and cheap food.	Limited to informal migrants in Dhaka city, small sample size
[39]	Cross-sectional survey, 106 urban and 106 rural households	Selling or crediting property, lending food and money, reducing food quality and amount	Lack of theoretical dimension, short study timeline
[40]	Gross margin analysis, n = 120	Disruption of supply chain, income loss, limiting access to market, and productional capacity, reduction in vegetables’ cultivation, declined food consumption	No theoretical dimension, only focused on vegetables supply and food security, farmers as respondents
[41]	Empirical work, n = 201	Shift in food intake and diet, drop in household income, stockpiling food, skipping food or reducing consumption, raising the amount of budget allotted to food, getting food aid, borrowing	Limited to nine weeks lockdown, no discussion on the role of NGO or government, no theoretical discussion

**Table 2:** Tabular-based literature review about COVID-19 induced food security on indigenous communities worldwide.

Reference/Sources	Study Area	Methodology and Sample Size	Key Findings	Limitations
[42]	Indonesia	Cross-sectional study with n = 517	Household Income loss, closure of work, high food insecurity in low-income families and houses comprising a younger member	Online interviews, less variation in socio- demographic profile, no theoretical implication, and inability to use conventional food consumption instruments
[43]	USA	Longitudinal study and Food Security Survey Module (FSSM) with n = 167	Women were more likely to have food insecurity, were less capable of affording proper meals, ended up eating smaller portions, and were more likely to starve than men	Lack of theoretical link, and missing discussion on Blackfeet tribal community’s coping strategies during COVID-19
[44]	USA	Cross-sectional study, n = 74,413	Households headed by Asian, Black, Hispanic, or other racial minorities were not remarkably more food insecure than White households	Main focus on food access, no discussion on the nutritional consequences of food insecurity, cross-sectional research design and week-one HPS microdata, absence of theoretical dimension
[45]	Arctic zone of Western Siberia	Multidisciplinary approach, n = 252	Insufficient access to local food, vaccines and medicines, rise in production cost, reduction in selling reindeer products’ price, change in food diet, health risk	Missing theoretical dimension, limited numbers of participants in reindeer herding industry
[46]	USA	Cross-sectional study, 3133 US counties	Infection rates were higher in Black, American Indian, or Alaska Native group with higher food scarcity and vast populations	Absence of theoretical framework, no explanation about food assistances or support services, and policy measures
[47]	India	Cross-sectional study n = 211	Barriers in getting ration, access to limited food items, starvation, lack of food supply	No theoretical framework, phone interview, short period

**Table 3:** Thematic data analysis procedure using Granheim and Lundman’s approach.

Steps	Description
1. Interview transcription	The interviews were taped and read again after hearing the recordings several times to comprehend their contents.
2. Unit for the formation of meaning analysis	All interviews were analyzed as a single unit. Primary codes were created by abstracting the meaning units.
3. Comprehensive sorting of similar codes	The grouping of similar fundamental codes into more comprehensive categories was conducted.
4. Comparison of codes and establishment of subcategories	In contrast, all codes and data identified similarities and differences. This process resulted in the formation of categories and subcategories.
5. Comparing subcategories and establishing primary categories	The initial interviews yielded an initial set of codes, categories, and subcategories, and the emerging codes were considered to be the results due to the thematic analysis approach.

**Table 4:** Defined themes that were derived from the thematic analysis.

Central Theme	Sub-Theme	Reference Code from NVivo-12	Descriptive Coding
Food Insecurity	Decreased consumption	88	*We had to reduce our consumption due to pandemic*
	Increase the price of daily food items	67	*The shop was closed, so we had to buy urgent goods at a high price secretly. So, isn’t that a problem?*
	Income shock to the food crisis	64	*We have no income right now. We hardly afford our budget for food due to our poor condition*
	Poor nutrition	41	*The most difficult time for a mother is when she cannot feed her kids. I could not eat food properly, so there was no milk in my breast*
	Shifting to unhealthy and inexpensive food	36	*This is so embarrassing, but the truth is that I had no money during the lockdown. So, I bought rotten vegetables and rice. Even*
	Starvation and hunger	29	*I cannot afford to have two-times meal. I am worried about Starvation*
	Food insufficiency leads to mental stress	28	*The sensation of hunger is affecting my child’s mental health*
Coping Strategies	Taking loans and borrowing foods	72	*I had to borrow about lakhs both from Janata bank and relatives. Having no income for such a long-time of 6/7 months, I had to take this money to run the family.*
	Reduced expenses and savings	60	*We used to eat fish meat almost every day, two days in a week. Now to deal with the situation, I do not buy fish anymore.*
	Changing food habits	49	*Where I used to eat three times, then I had to eat twice a day with one kg of pulses.*
	Collecting forest and hill foods	44	*To feed my family, I collect beans, green jack fruits, seeds from the forest.*
	Selling domestic animals and property	29	*Before, I had three tomtoms; now, there is only one. I had to sell the other two due to the food crisis.*
	Social and governmental reliefs	25	*Apart from the chairman’s members, an organization was also cooperating from other places. I don’t know which organization it is. Besides, some rich people helped us.*

**Table 5:** Distribution of the demographic profile of the interviewees.

Category	Variable	N
Gender	Men	39
	Women	21
Age	20–30	17
	30–40	15
	40–50	11
	50–60 60–70	10 7
Education	Illiterate	32
	Under Primary School	13
	Primary School	9
	High School	6
Marital status	Married	46
	Widow	14
Ethnicity	Chakma	24
	Marma	18
	Tripura	9
	Tanchangya	9
Place of residence	Khagrachari	20
	Rangamati	20
	Bandarban	20

## References

[1] Chriscaden K. (2020). Impact of COVID-19 on People’s Livelihoods, Their Health and Our Food Systems.

[2] Sohel M.S., Hossain B., Sarker M.N.I., Horaira G.A., Sifullah M.K., Rahman M.A. (2021). Impacts of COVID-19 induced food insecurity among informal migrants: Insight from Dhaka, Bangladesh. J. Public Aff..

[3] World Food Program (2020). WFP Global Update on COVID-19: November 2020.

[4] WFP (2021). COVID-19 Will Double Number of People Facing Food Crises Unless Swift Action Is Taken.

[5] Oxfam International (2021). A Rapid Assessment Report: The impact of COVID-19 on Indigenous and Tribal Peoples in Bangladesh.

[6] United Nations (2020). Food & United Nations.

[7] (2021). Africa Hunger, Famine: Facts, FAQs, and How to Help. In World Vision. https://www.worldvision.org/hunger-news-stories/africa-hunger-famine-facts.

[8] Rashid S.F., Theobald S., Ozano K. (2020). Towards a socially just model: Balancing hunger and response to the COVID-19 pandemic in Bangladesh. BMJ Glob. Health.

[9] Pereira M., Oliveira A.M. (2020). Poverty and food insecurity may increase as the threat of COVID-19 spreads. Public Health Nutr..

[10] Bukari C., Aning-Agyei M.A., Kyeremeh C., Essilfie G., Amuquandoh K.F., Owusu A.A., Otoo I.C., Bukari K.I. (2021). Effect of COVID-19 on Household Food Insecurity and Poverty: Evidence from Ghana. Soc. Indic. Res..

[11] Kansiime M.K., Tambo J.A., Mugambi I., Bundi M., Kara A., Owuor C. (2020). COVID-19 implications on household income and food security in Kenya and Uganda: Findings from a rapid assessment. World Dev..

[12] El Bilali H., Ben Hassen T., Baya Chatti C., Abouabdillah A., Alaoui S.B. (2021). Exploring Household Food Dynamics during the COVID-19 Pandemic in Morocco. Front. Nutr..

[13] Holt L., Murray L. (2021). Children and Covid 19 in the UK. Taylor Fr..

[14] Simmet A., Stroebele-Benschop N. (2021). Food Bank Operations during the COVID-19 Pandemic in Germany. J. Hunger Environ. Nutr..

[15] Buchholz K. (2021). How Has COVID-19 Impacted Food Security in the US? World Economic Forum. https://www.weforum.org/agenda/2021/09/united-states-us-food-insecurity-covid-pandemic-lockdown-coronavirus/.

[16] Dondi A., Candela E., Morigi F., Lenzi J., Pierantoni L., Lanari M. (2020). Parents’ Perception of Food Insecurity and of Its Effects on Their Children in Italy Six Months after the COVID-19 Pandemic Outbreak. Nutrients.

[17] Golunov S., Smirnova V. (2021). Russian Border Controls in Times of the COVID-19 Pandemic: Social, Political, and Economic Implications. Probl. Post-Communism.

[18] Fanelli R.M. (2021). Changes in the food-related behaviour of italian consumers during the covid-19 pandemic. Foods.

[19] Chan L. (2019). Final Report for Eight Assembly of First Nations Regions. Assembly of First Nations, University of Ottawa, Université de Montréal, Canada. https://www.fnfnes.ca/docs/FNFNES_draft_technical_report_Nov_2__2019.pdf.

[20] Cupertino G.A., do Carmo Cupertino M., Gomes A.P., Braga L.M., Siqueira-Batista R. (2020). COVID-19 and Brazilian indigenous populations. Am. J. Trop. Med. Hyg..

[21] UN (2021). UN/DESA Policy Brief #70: The Impact of COVID-19 on Indigenous Peoples. Department of Economic and Social Affairs. https://www.un.org/development/desa/dpad/publication/un-desa-policy-brief-70-the-impact-of-covid-19-on-indigenous-peoples/.

[22] ILO (2020). COVID-19 and the world of work: A focus on indigenous and tribal peoples, Policy Brief. Pancanaka.

[23] Roy P., Deshwara M., Ethnic Population in 2022 Census: Real Picture Not Reflected. The Daily Star (2022). https://www.thedailystar.net/news/bangladesh/news/ethnic-population-2022-census-real-picture-not-reflected-3090941.

[24] Jamil I., Panday P.K. (2008). The elusive peace accord in the Chittagong Hill Tracts of Bangladesh and the plight of the indigenous people. Commonw. Comp. Polit..

[25] Faruk M.O., Ching U., Chowdhury K.U.A. (2021). Mental health and well-being of indigenous people during the COVID-19 pandemic in Bangladesh. Heliyon.

[26] Rasul G. (2015). A Strategic Framework for Sustainable Development in the Chittagong Hill Tracts of Bangladesh.

[27] Ali H.M.A., Vallianatos H. (2017). Women’s Experiences of Food Insecurity and Coping Strategies in the Chittagong Hill Tracts, Bangladesh. Ecol. Food Nutr..

[28] Barkat A., Halim S. (2009). Baseline Survey of Chittagong Hill Tracts. Methodology. UNDP. http://www.hdrc-bd.com/wp-content/uploads/2018/12/6.-Socio-economic-Baseline-Survey-of-Chittagong-Hill-Tracts.pdf.

[29] Kirstyn M. (2021). Covid-19 Exacerbates Food Insecurity for Indigenous Peoples. International Bar Association. https://www.ibanet.org/article/D36C40DA-C2CA-4E1C-94AB-557CB1BF648F.

[30] Shibli A. (2021). Food Insecurity Increases Amidst the Latest Covid-19 Spike. https://www.thedailystar.net/opinion/open-dialogue/news/food-insecurity-increases-amidst-the-latest-covid-19-spike-2087621.

[31] Trimita Chakma P.C. (2020). Still Left behind: Covid-19 and Indigenous Peoples of Bangladesh. The Daily Star. https://www.thedailystar.net/opinion/news/still-left-behind-covid-19-and-indigenous-peoples-bangladesh-1941817.

[32] Rabbi M.F., Oláh J., Popp J., Máté D., Kovács S. (2021). Food security and the covid-19 crisis from a consumer buying behaviour perspective—The case of Bangladesh. Foods.

[33] Mottaleb K.A., Mainuddin M., Sonobe T. (2020). COVID-19 induced economic loss and ensuring food security for vulnerable groups: Policy implications from Bangladesh. PLoS ONE.

[34] Mobarok M.H., Thompson W., Skevas T. (2021). COVID-19 and policy impacts on the Bangladeshi rice market and food security. Sustainability.

[35] Mueller V., Grépin K.A., Rabbani A., Navia B., Ngunjiri A.S.W., Wu N. (2022). Food insecurity and COVID-19 risk in low- and middle-income countries. Appl. Econ. Perspect. Policy.

[36] Ahmed F., Islam A., Pakrashi D., Rahman T., Siddique A. (2021). Determinants and dynamics of food insecurity during COVID-19 in rural Bangladesh. Food Policy.

[37] Mandal S.C., Boidya P., Haque M.I.M., Hossain A., Shams Z., Mamun A. (2021). Al The impact of the COVID-19 pandemic on fish consumption and household food security in Dhaka city, Bangladesh. Glob. Food Sec..

[38] Kundu S., Al Banna M.H., Sayeed A., Sultana M.S., Brazendale K., Harris J., Mandal M., Jahan I., Abid M.T., Khan M.S.I. (2021). Determinants of household food security and dietary diversity during the COVID-19 pandemic in Bangladesh. Public Health Nutr..

[39] Das S., Rasul M.G., Hossain M.S., Khan A.-R., Alam M.A., Ahmed T., Clemens J.D. (2020). Acute food insecurity and short-term coping strategies of urban and rural households of Bangladesh during the lockdown period of COVID-19 pandemic of 2020: Report of a cross-sectional survey. BMJ Open.

[40] Alam G.M.M., Khatun M.N. (2021). Impact of COVID-19 on vegetable supply chain and food security: Empirical evidence from Bangladesh. PLoS ONE.

[41] Ruszczyk H.A., Rahman M.F., Bracken L.J., Sudha S. (2021). Contextualizing the COVID-19 pandemic’s impact on food security in two small cities in Bangladesh. Environ. Urban..

[42] Syafiq A., Fikawati S., Gemily S.C. (2022). Household food security during the COVID-19 pandemic in urban and semi-urban areas in Indonesia. J. Health Popul. Nutr..

[43] John-Henderson N.A., Oosterhoff B.J., Johnson L.R., Ellen Lafromboise M., Malatare M., Salois E. (2022). COVID-19 and food insecurity in the Blackfeet Tribal Community. Food Secur..

[44] Morales D.X., Morales S.A., Beltran T.F. (2020). Racial/Ethnic Disparities in Household Food Insecurity During the COVID-19 Pandemic: A Nationally Representative Study. J. Racial Ethn. Health Disparities.

[45] Bogdanova E., Andronov S., Morell I.A., Hossain K., Raheem D., Filant P., Lobanov A. (2020). Food Sovereignty of the Indigenous Peoples in the Arctic Zone of Western Siberia: Response to COVID-19 Pandemic. Int. J. Environ. Res. Public Health.

[46] Kimani M.E., Sarr M., Cuffee Y., Liu C., Webster N.S. (2021). Associations of race/ethnicity and food insecurity with COVID-19 infection rates across US counties. JAMA Netw. Open.

[47] Saxena A., Mohan S.B. (2021). The impact of food security disruption due to the Covid-19 pandemic on tribal people in India. Advances in Food Security and Sustainability.

[48] Salin M., Kaittila A., Hakovirta M., Anttila M. (2020). Family coping strategies during finland’s COVID-19 lockdown. Sustainability.

[49] Helms H.M., Supple A.J., Proulx C.M. (2011). Mexican-Origin Couples in the Early Years of Parenthood: Marital Well-Being in Ecological Context. J. Fam. Theory Rev..

[50] Mantel S., Khan M. Chittagong Hill Tracts Improved Natural Resource Management. Proceedings of the National Workshop held in Rangamati.

[51] Sandelowski M. (1995). Qualitative analysis: What it is and how to begin. Res. Nurs. Health.

[52] Crouch M., McKenzie H. (2006). The logic of small samples in interview-based qualitative research. Soc. Sci. Inf..

[53] Black K. (2010). Business Statistics For Contemporary Decision Making.

[54] Dawson S., Manderson L., Tallo V.L. (1993). Methods for Social Research in Disease. A Manual for the Use of Focus Groups.

[55] Saunders M., Lewis P., Thornhill A. (2009). Research Methods for Business Students.

[56] Berg B.L. (2012). Qualitative Research Methods for the Social Sciences.

[57] Denzin N. (2010). An Introduction to Triangulation.

[58] Graneheim U.H., Lundman B. (2004). Qualitative content analysis in nursing research: Concepts, procedures and measures to achieve trustworthiness. Nurse Educ. Today.

[59] Yin R.K. (2011). Qualitative Research from Start to Finish.

[60] Elo S., Kääriäinen M., Kanste O., Pölkki T., Utriainen K., Kyngäs H. (2014). Qualitative Content Analysis. SAGE Open.

[61] Muellbauer J. (2020). The Coronavirus Pandemic and US Consumption.VOX, CEPR, Centre for Economic Policy Research. https://cepr.org/voxeu/columns/coronavirus-pandemic-and-us-consumption.

[62] Pan D., Yang J., Zhou G., Kong F. (2020). The influence of COVID-19 on agricultural economy and emergency mitigation measures in China: A text mining analysis. PLoS ONE.

[63] Yuit C.K., On P.A. (2021). Counting the Cost as New COVID-19 Waves Disrupt Supply Chains in Asia.

[64] UNCTAD Shipping during COVID-19: Why Container Freight Rates Have Surged; United Nations Conference on Trade and Development: 2021. Geneva, Switzerland. https://unctad.org/news/shipping-during-covid-19-why-container-freight-rates-have-surged.

[65] Akter S., Basher S.A. (2014). The impacts of food price and income shocks on household food security and economic well-being: Evidence from rural Bangladesh. Glob. Environ. Chang..

[66] Islam A., Rahman A., Nisat R. (2020). The Impact of COVID-19 Pandemic on Small and Medium Enterprises in Bangladesh.

[67] (2016). Malnutrition Hangs on Hill Districts. The Asian Age. https://dailyasianage.com/news/42994/malnutrition-hangs-on-hill-districts.

[68] The World Bank (2022). Food Security and COVID-19.

[69] Hansen T. How Covid-19 Could Destroy Indigenous Communities-BBC Future. :https://www.bbc.com/future/article/20200727-how-covid-19-could-destroy-indigenous-communities.

[70] Reid K. (2022). 10 World Hunger Facts You Need to Know. https://www.worldvision.org/hunger-news-stories/world-hunger-facts.

[71] Al Jazeera World Hunger Rising as UN Agencies Warn of ‘Looming Catastrophe’. https://www.aljazeera.com/news/2022/7/6/world-hunger-rising-as-un-agencies-warn-of-looming-catastrophe.

[72] Rozario S.U. (2020). Ethnic Communities Face Starvation in Bangladesh. https://www.ucanews.com/news/ethnic-communities-face-starvation-in-bangladesh/87768.

[73] Fang D., Thomsen M.R., Nayga R.M. (2021). The association between food insecurity and mental health during the COVID-19 pandemic. BMC Public Health.

[74] Hossain B., Shi G., Ajiang C., Sarker N.I., Sohel S., Sun Z., Hamza A. (2021). Impact of climate change on human health: Evidence from riverine island dwellers of Bangladesh. Int. J. Environ. Health Res..

[75] Hossain B., Ryakitimbo C.M., Sohel M.S. (2020). Climate Change Induced Human Displacement in Bangladesh: A Case Study of Flood in 2017 in Char in Gaibandha District. Asian Res. J. Arts Soc. Sci..

[76] Hossain B., Sohel M.S., Ryakitimbo C.M. (2020). Climate change induced extreme flood disaster in Bangladesh: Implications on people’s livelihoods in the Char Village and their coping mechanisms. Prog. Disaster Sci..

[77] Hossain B., Sarker N.I., Sohel S. (2021). Assessing the Role of Organizations for Health Amenities of Flood Affected People in Char Areas of Bangladesh.

[78] Huston T.L. (2000). The social ecology of marriage and other intimate unions. J. Marriage Fam..

[79] Hill A. (2020). “One Meal a Day”: How Pandemic Hit Families before Unicef’s Aid. The Guardian. https://www.theguardian.com/society/2020/dec/18/one-meal-a-day-how-pandemic-hit-families-before-unicefs-aid.

[80] Sohel M.S., Hossain B., Alam M.K., Shi G., Shabbir R., Sifullah M.K., Mamy M.M.B. (2021). COVID-19 induced impact on informal migrants in Bangladesh: A qualitative study. Int. J. Sociol. Soc. Policy.

[81] Ashraf Ali H.M., Vallianatos H. (2016). Indigenous foodways in the Chittagong Hill Tracts of Bangladesh: An alternative-additional food network. Postcolonialism, Indigenity and Struggles for Food Sovereignty: Alternative Food Networks in Subaltern Spaces.

[82] Rahman M.H., Alam K. (2016). Forest dependent indigenous communities’ perception and adaptation to climate change through local knowledge in the protected area-A Bangladesh Case Study. Climate.

[83] Miah M.D., Chakma S., Koike M., Muhammed N. (2012). Contribution of forests to the livelihood of the Chakma community in the Chittagong Hill Tracts of Bangladesh. J. For. Res..

[84] Madden C. (2021). Indigenous Food Systems Prove Highly Resilient during COVID-19.

[85] Leweniqila I., Vunibola S. (2020). Food Security in COVID-19: Insights from Indigenous Fijian Communities. Oceania.

[86] Pakravan-Charvadeh M.R., Savari M., Khan H.A., Gholamrezai S., Flora C. (2021). Determinants of household vulnerability to food insecurity during COVID-19 lockdown in a mid-term period in Iran. Public Health Nutr..

[87] Cappelli A., Cini E. (2020). Will the COVID-19 pandemic make us reconsider the relevance of short food supply chains and local productions?. Trends Food Sci. Technol..

[88] (2020). Food in a time of COVID-19. Nat. Plants.

[89] Akter S. (2020). The impact of COVID-19 related ‘stay-at-home’ restrictions on food prices in Europe: Findings from a preliminary analysis. Food Secur..

[90] United Nations (2020). The Impact of COVID-19 on Indigenous Peoples. https://www.un.org/development/desa/dpad/wp-content/uploads/sites/45/publication/PB_70.pdf.

[91] Hossain B., Shi G., Ajiang C., Sarker M.N.I., Sohel M.S., Sun Z., Yang Q. (2022). Climate change-induced human displacement in Bangladesh: Implications on the livelihood of displaced riverine island dwellers and their adaptation strategies. Front. Psychol..

[92] Saxena A., Amin A., Mohan S.B., Mohan P. (2020). Food Insecurity in Tribal High Migration Communities in Rajasthan, India. Food Nutr. Bull..

[93] (2021). Indigenous Food Systems Are at the Heart of Resilience.

[94] Levkoe C.Z., McLaughlin J., Strutt C. (2021). Mobilizing Networks and Relationships Through Indigenous Food Sovereignty: The Indigenous Food Circle’s Response to the COVID-19 Pandemic in Northwestern Ontario. Front. Commun..

[95] (2020). Colombia: Indigenous Kids at Risk of Malnutrition, Death.

[96] Galali Y. (2021). The impact of COVID-19 confinement on the eating habits and lifestyle changes: A cross sectional study. Food Sci. Nutr..

[97] Hirvonen K., de Brauw A., Abate G.T. (2021). Food Consumption and Food Security during the COVID-19 Pandemic in Addis Ababa. Am. J. Agric. Econ..

[98] Kaplan J. (2020). 14 Countries that Are Paying Their Workers during Quarantine—And How They Compare to America’s $1200 Stimulus Checks. Insider. https://www.businessinsider.com/countries-offering-direct-payments-or-basic-income-in-corona-crisis-2020-4.

[99] Sohel S., Azimul S., Noshin E., Zaman T. (2022). Understanding rural local government response during COVID-19-induced lockdown: Perspective from Bangladesh. SN Soc. Sci..

